# Caring over computing: an ethical and sociotechnical perspective on digital technologies for social connectedness in dementia care

**DOI:** 10.3389/fdgth.2026.1944047

**Published:** 2026-09-09

**Authors:** Teis Arets, Giulia Perugia, Maarten Houben, Wijnand IJsselsteijn

**Affiliations:** 1 Human Technology Interaction, Eindhoven University of Technology, Eindhoven, Netherlands; 2 Department of Industrial Design, Eindhoven University of Technology, Eindhoven, Netherlands

**Keywords:** care professionals, dementia, digital technologies, ethics, social connectedness, sociotechnical systems

## Abstract

People with dementia in residential care often experience reduced social connectedness. Person-centered care approaches foreground meaningful social interactions to support well-being, but staff and time pressures increasingly constrain opportunities for such care. Digital technologies offer possibilities to help care professionals facilitate social connectedness, despite increasing care pressures. Yet, the meaningful integration of such technologies into the complex sociotechnical setting of residential dementia care is not straightforward. To responsibly design and position digital technologies for dementia care, this study empirically explores care professionals’ perspectives on social connectedness and the sociotechnical role of conventional digital technologies in residential dementia care. We conducted five workshops with 15 care professionals from three care organizations to pursue this objective. Our findings show that fostering social connectedness is a relational process involving continuous interpretation, negotiation, and adaptation to residents’ individual needs, personal values, and institutional constraints. Digital technologies were used by care professionals to support communication, interaction, and shared activities, yet remained largely peripheral to everyday care routines and were primarily used reactively in response to observed behavior. Drawing on an ethics of care framework, we derive ethical and sociotechnical implications for the responsible design and positioning of digital technologies in residential dementia care.

## 1 Introduction

Dementia refers to neurodegenerative conditions characterized by progressive decline in cognition, physical abilities, and psychosocial well-being (1, 2), affecting over 55 million people worldwide. Dementia directly impacts social health, reducing opportunities for social interaction, diminishing the ability to participate in social activities (3), and increasing the difficulty of following and sustaining everyday interactions (4, 5). These challenges limit opportunities to experience social connectedness, the sense of belonging to a social group or network (6, 7), especially upon admission to residential care (8).

Person-centered dementia care approaches recognize social connectedness as central to the well-being and personhood of people with dementia (9, 10). In the context of person-centered dementia care, care professionals are positioned as primary actors in fostering social connectedness, not only because of their continuous presence, but also due to their role in facilitating everyday interaction and supporting residents’ socio-emotional well-being (11). Yet, the enactment of person-centered dementia care increasingly takes place under conditions of staff shortages (12), workload and emotional strain among care professionals (13), and time-constrained care practices within residential care environments (14, 15). These conditions restrict opportunities for meaningful social interaction and engagement, despite the recognized importance of the latter for the well-being of people with dementia.

In recent years, the field of Human-Computer Interaction (HCI) has increasingly explored how digital technologies can support social connectedness among people with dementia in care contexts (16, 17). For instance, digital technologies have been used to facilitate shared experiences through multi-user Virtual Reality (VR) (18), support social interaction by letting people with dementia receive and respond to messages and media and share their own creations and experiences (19–21), and foster social interaction through collaborative activities using technology-based open-ended play (22). Emerging AI-based technologies have further been explored for supporting companionship, reminiscence, and storytelling (23–25). However, the extent to which such technologies can foster meaningful social connectedness is shaped not only by their technical affordances, but also by how they are incorporated into everyday care practices (26). Their use may require care professionals to facilitate interactions, adapt technologies to residents’ needs and abilities, and integrate them into existing routines and relationships, highlighting that digital technologies are embedded within, and may reshape, complex sociotechnical care practices (26–28). Thus, the introduction of digital technologies into dementia care for social connectedness raises not only practical questions about how such systems can be developed for this context, but also ethical and sociotechnical questions about how they can be responsibly positioned within the relational processes through which care professionals recognize and enact social connectedness in practice. To understand these ethical and sociotechnical implications, it is therefore important to examine how care professionals understand and foster social connectedness in everyday care practice, as well as how they understand the role of digital technologies within these practices. To this end, we pose the following research questions:

**RQ1** How do care professionals understand and approach fostering social connectedness for people with dementia in residential care?
**RQ2** How do care professionals understand the role of digital technologies in fostering social connectedness in residential dementia care?

We draw on Tronto’s (29) ethics of care framework to structure our Discussion and reflect on our findings. This paper makes three contributions: (1) it describes how care professionals recognize, negotiate, and enact social connectedness in residential care; (2) it conceptualizes social connectedness as a relational care process through which social needs are continuously interpreted and enacted; and (3) it derives ethical and sociotechnical considerations for the responsible positioning of digital technologies for promoting social connectedness in dementia care.

## 2 Background

### 2.1 Social connectedness in dementia care

Social connectedness is associated with factors such as relationship salience, closeness, shared understanding, and quality of social contact (7). The experience of social connectedness positively affects quality of life and mental well-being (30, 31). However, people with dementia often experience reduced opportunities for social connectedness, particularly in residential care settings, as cognitive and physical decline limit communication abilities (5, 32, 33) and opportunities to engage in social interaction (34). As a result, many people with dementia live with unmet social needs (35–37), which are often expressed indirectly through so-called responsive behaviors (35, 36, 38, 39). In residential care, care professionals play a central role in interpreting such responsive behavior and translating it into social needs that can be acted upon (37). Accordingly, care professionals often rely on familiarity, observation, and knowledge developed through ongoing everyday interactions to recognize social connectedness needs (40).

At the same time, HCI is increasingly exploring how digital technologies can support social connectedness in dementia care (16, 17). To ensure that such technologies can be meaningfully integrated into everyday care practices, it is important to understand how care professionals recognize and foster social connectedness and how technology can be situated within these practices.

### 2.2 Digital technologies for social connectedness

Digital technologies have been explored in HCI to support social connectedness among people with dementia through diverse forms of communication, shared experience, and collaborative activity. For instance, digital technologies can support social connectedness by translating personalized images, artwork, and other digital content into tangible media through which people with dementia can choose what to communicate, with whom, and how, supporting social interaction in ways that accommodate their preferences and capacities (19–21). Furthermore, emerging Generative AI-based technologies can support social interaction through activities such as reminiscence and storytelling, with people with dementia actively shaping the interaction by contributing prompts, selecting or responding to generated content, and co-constructing stories with others (23–25, 41, 42). Similarly, technology-based open-ended play fosters social interactions between people with dementia by engaging them in self-directed, collaborative activities (22). Finally, multi-user VR can support social connectedness between people with dementia and their social contacts by enabling them to jointly explore familiar environments and engage in shared experiences, using personally meaningful places as a basis for interaction (18). Across these approaches, technology is not necessarily positioned as a substitute for social interaction, but as a medium through which people with dementia can participate in, shape, and contribute to interactions with others.

However, these technological possibilities do not exist independently of the contexts in which they are introduced. The introduction of digital technologies into residential care with a social purpose inevitably introduces a sociotechnical disruption that reconfigures care practice by reshaping routines, interactions, actors, and responsibilities in everyday care work (27, 28, 43). Research on technology use in residential care similarly shows that adoption and non-use are shaped by situated factors such as staff practices, organizational conditions, available resources, and the perceived relevance of technologies to everyday care (44–46). Care professionals are therefore not merely users of these technologies, but important mediators of how they become incorporated into care practices: they may facilitate technology-mediated interactions, determine when and how technologies are used, and adapt their use to residents’ needs and the demands of particular situations (26). Consequently, the potential of a technology to support social connectedness cannot be understood solely from its technical affordances, but needs to be considered in relation to the sociotechnical context in which care professionals and people with dementia encounter and use it.

Despite emerging work exploring digital technologies for social interaction, little is known about how care professionals understand and position such technologies within the relational processes through which social connectedness is recognized and enacted. One line of work has explored person-centered practices in dementia care from the perspective of care professionals [e.g., (9, 40, 47)], while another has examined how technologies may support care professionals in practice [e.g., (17, 45, 46)]. However, there remains a gap in bringing these lines of research together to examine how digital technologies may fit within care professionals’ ongoing person-centered practices. This raises critical questions not only about what such technologies *can* do, but about where, how, and whether they *should* be integrated into the process of fostering social connectedness. In this paper, we address this gap by investigating how care professionals perceive and enact social connectedness in practice with and without the support of technologies, and position digital technologies within this process. These considerations extend beyond technological functionality to raise ethical and sociotechnical questions about how digital technologies can be appropriately integrated into dementia care while supporting the relational practice of fostering social connectedness.

### 2.3 Ethical perspectives on social connectedness and digital technologies

To understand how care professionals may foster social connectedness, it is important to consider the processes through which caregiving takes place. Care in dementia is not a clear-cut process but involves dynamic and individualized responses to the needs, preferences, and circumstances of the person receiving care (9, 10, 40). To conceptualize these relational processes, we draw on Tronto’s (29) ethics of care framework–a relational sociotechnical perspective on caregiving that positions care as grounded in relationships, responsibility, and situated responses to the needs of others, rather than abstract moral rules (44, 48, 49). Through the ethics of care framework, we can conceptualize fostering social connectedness as a relational care practice through an ethics of care lens. Tronto conceptualizes care as comprising four interconnected phases: *caring about*, which involves recognizing a need; *taking care of*, which involves assuming responsibility for addressing that need; *care giving*, which concerns the enactment of care; and *care receiving*, which attends to how care is received and whether the need has been met (29). Applied to social connectedness, the framework provides a way of conceptualizing how social needs may be recognized, responded to, enacted, and evaluated as part of ongoing care. An important caveat to this framework is that in practice, the phases of care are never as linear as they appear here (29). Nevertheless, this ethics of care framework helps us make the practices that foster social connectedness visible and evaluate the positioning of digital technologies within the relational processes through which social needs are recognized and addressed in residential dementia care.

When digital technologies become part of these care processes, they may affect, transform, or challenge the relationships and practices through which care is enacted (26, 50–53). Recent work on technology integration in geriatric care characterizes this as a potential “double-edged sword”: technologies may offer benefits such as greater efficiency, safety, and support for care, while simultaneously creating tensions with values including autonomy, dignity, equity, and human connection (50, 51). Ramvi et al. similarly highlight that, although care remains fundamentally relational when mediated by technology, introducing technology can change how care relationships are enacted and create new ethical tensions within those relationships (52). In institutional dementia care, these tensions are further shaped by the different values and needs of those involved in care. In line with this, scholars have called for value-based technologies that not only ensure technical feasibility but also account for relational values such as caring and empathy, community, social inclusion, and individuality, alongside values such as autonomy and privacy that may come into tension with them (54). These perspectives suggest that the ethical implications of digital technologies cannot be considered independently of the relationships, values, and practices within which they are introduced.

In this study, we engage with Tronto’s ethics of care framework as a broader theoretical perspective to interpret and evaluate how social connectedness is navigated in practice. Rather than applying the framework as an a priori template for data collection or initial coding, we draw upon its four dimensions retrospectively as an interpretive lens to synthesize, contextualize, and discuss the ethical and sociotechnical implications emerging from our inductive empirical findings.

## 3 Materials and methods

### 3.1 Study design

We conducted five interactive workshops with care professionals to understand their perspectives on the social connectedness needs of people with dementia living in residential settings, and the use of technological interventions in dementia care. The study was approved by the ethical review board of the Human-Technology Interaction group at Eindhoven University of Technology (Approval number: 1978).

### 3.2 Participants and recruitment

Fifteen care professionals (13 women, 2 men) working with people with dementia, divided into groups of 2 to 4 (see Table 1 for an overview of the group composition), participated in the workshops. We aimed for three participants per workshop to facilitate interaction while allowing sufficient opportunity for each participant to contribute. For workshop 4, one recruited participant was unable to attend, resulting in two participants. For workshop 5, we had initially planned two workshops but recruited only four participants in total, who we therefore brought together in a single session.

**Table 1:** Workshop participants overview.

Code	Workshop	Organization	Gender	Age	Experience	Profession
P1	W1	A	F	30–39	3–5	Certified care assistant
P2	W1	A	F	50–59	5–10	Primary care coordinator
P3	W1	A	F	40–49	20+	Nurse with coordinating tasks
P4	W2	A	F	30–39	5–10	Certified care assistant
P5	W2	A	F	50–59	20+	Residential support worker
P6	W2	A	F	40–49	3–5	Certified care assistant
P7	W3	B	M	30–39	5–10	Social worker
P8	W3	B	F	20–29	1–2	Social worker
P9	W3	B	F	50–59	10–20	Certified care assistant
P10	W4	B	F	40–49	20+	Nurse
P11	W4	B	F	30–39	5–10	Psychologist
P12	W5	C	F	40–49	20+	Nurse with coordinating tasks
P13	W5	C	F	50–59	5–10	Certified care assistant
P14	W5	C	F	50–59	10–20	Nurse
P15	W5	C	M	50–59	20+	Advisor care & technology

Participants were recruited digitally by sharing information about the workshops on their organizations' intranets, central communication platforms, or through team leads. The care professionals who participated in the study were employed by three different care organizations (care homes A, B, and C, respectively employing P1–P6, P7–P11, and P12–P15; see Table 1). Each workshop consisted exclusively of professionals from the same organization. Due to the large size of these organizations, this did not necessarily imply that participants knew each other prior to the study or worked together. The workshops took place *in situ* at each care organization.

Most care professionals in our study worked with people in the mid to late stages of dementia. Our sample included a diverse range of professional roles, some familiar to readers worldwide (i.e., psychologist, nurse, social worker, advisor care and technology), others typical of the care context in which the data were collected. The latter are described below. *Certified care assistants* provide daily hands-on support to people with dementia in residential care, delivering personal care and basic nursing assistance. *Primary care coordinators* focus on maintaining ongoing, direct contact with a designated resident in residential care, supporting daily structure and meaningful engagement while serving as the primary liaison with relatives and the multidisciplinary team. *Residential support workers* support dementia care teams from a unit-wide perspective, coaching staff and setting up meaningful daily activities.

### 3.3 Workshop procedure

The workshops started with an introduction, during which the moderators explained to participants their rights (e.g., voluntary participation and withdrawal) and data handling procedures, and obtained written informed consent, after which they started the audio and video recordings.

**Figure 1:**
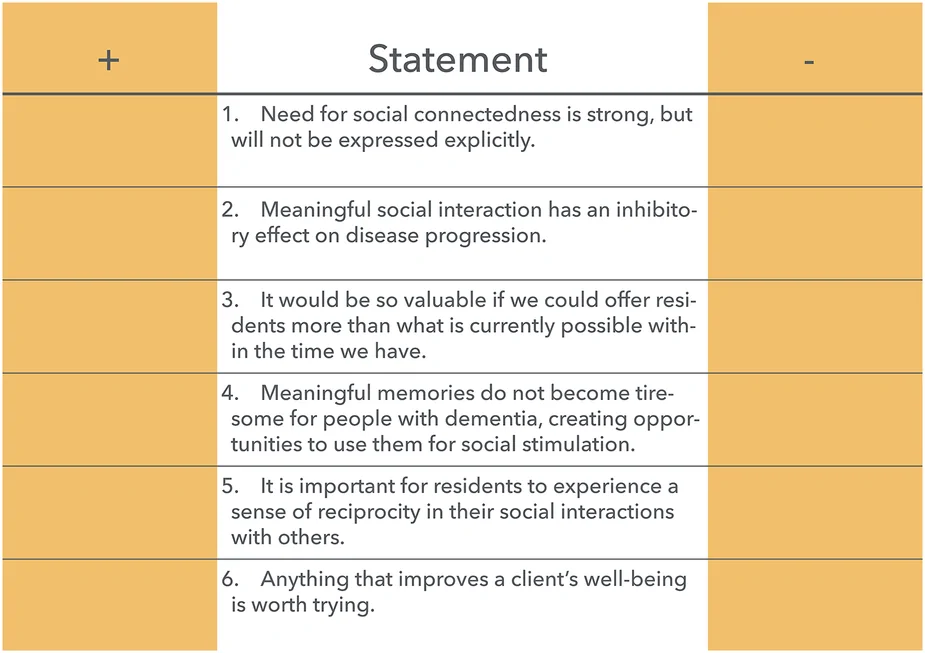
The sheet used during the first phase of the workshops.

**Figure 2:**
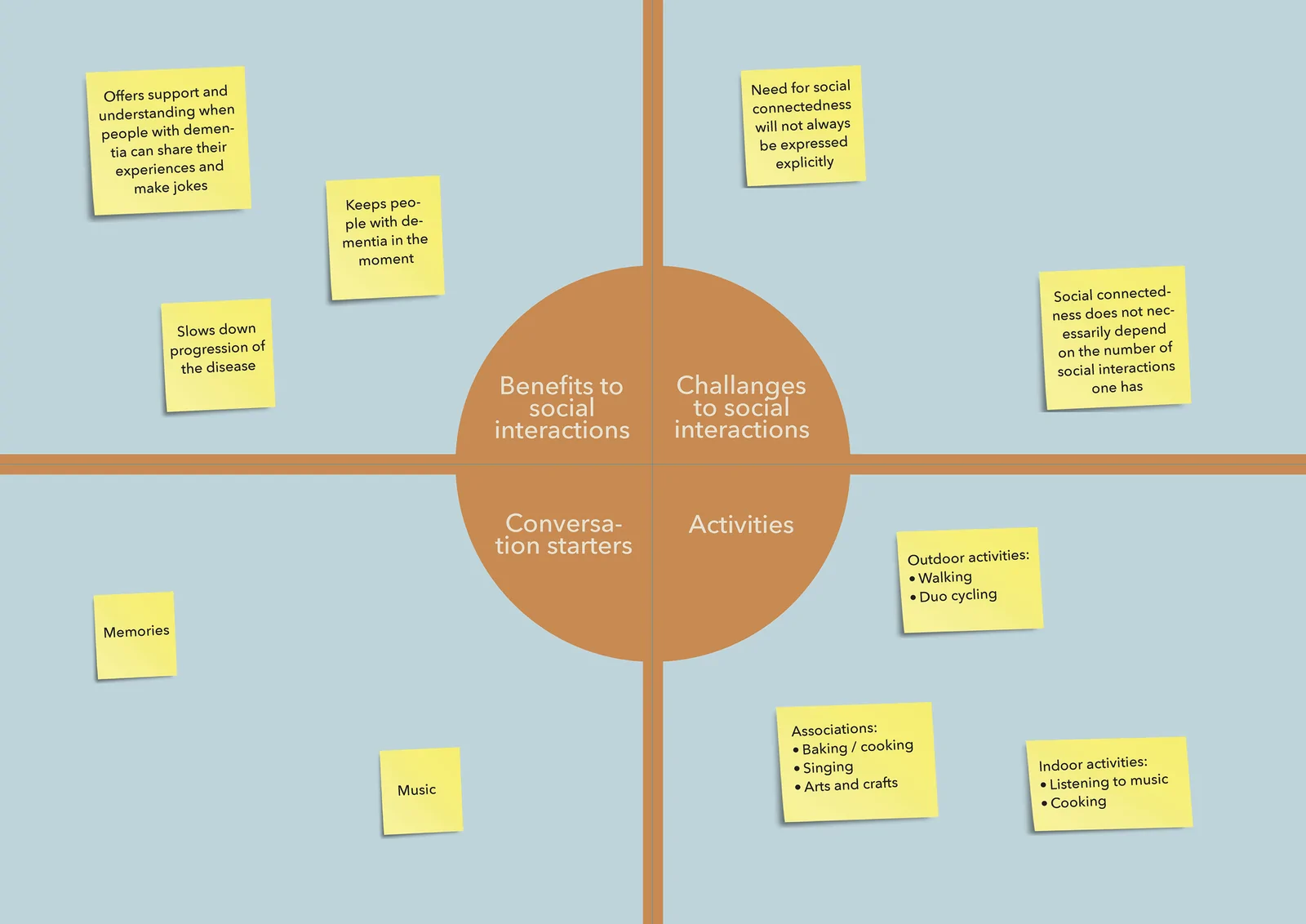
The sheet used during the second phase of the workshops.

**Figure 3:**
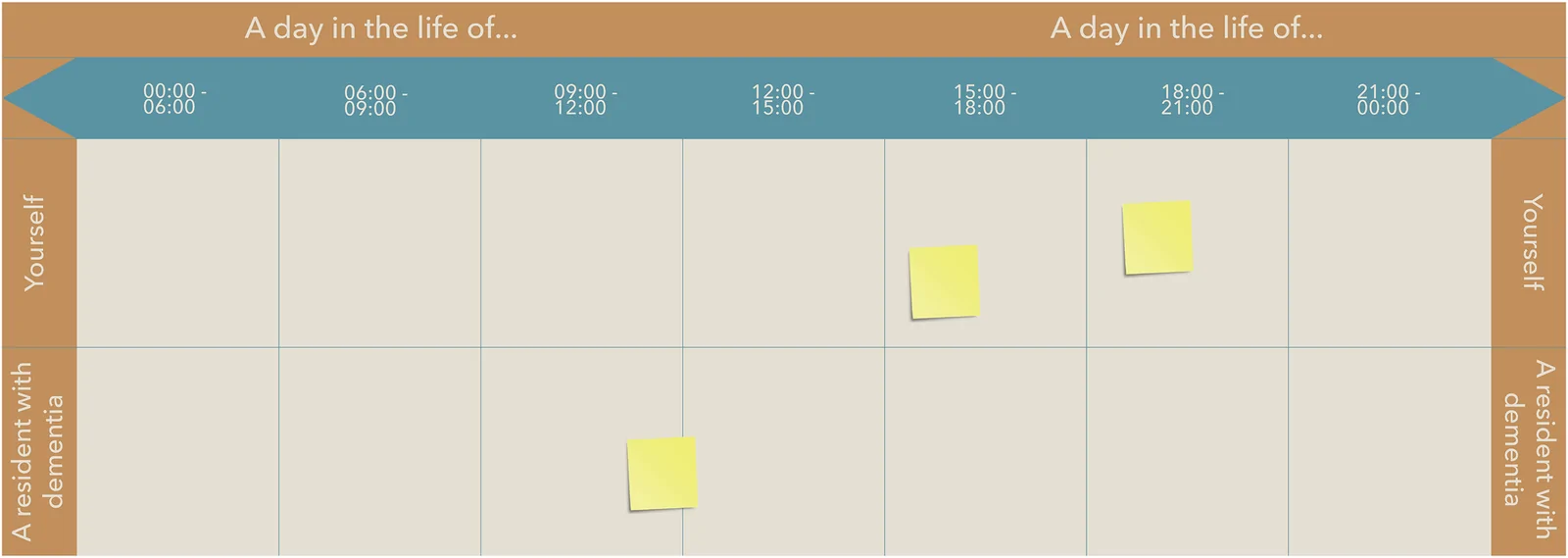
The sheet used during the third phase of the workshops.

The session consisted of three consecutive phases, each supported by the respective workshop materials in Figures 1–3. In the *first* phase, participants were presented with a table containing six statements on the social connectedness of people with dementia. They first individually reflected on whether they agreed or disagreed with the statements in the table, making notes on post-its, then discussed their reflections with the group. In the *second* phase, participants used a poster with four quadrants: (1) benefits of social interactions, (2) challenges of social interactions, (3) conversation starters, and (4) activities with a social component. They collaboratively explored each quadrant by adding post-its to the poster and engaging in a group discussion on each other’s experiences and insights. In the *third* phase, participants employed an empty template to map a day in their life and in the life of a person with dementia. They used post-its to describe daily tasks and activities, creating a situated representation of how care unfolds in practice, and reflecting on opportunities for social stimulation.

As a closing discussion topic, participants reflected on their experiences around technology use in dementia care, and provided their perspectives on how technologies are currently used in relation to social connectedness. After the workshop, participants received a gift worth €12.

#### 3.3.1 Workshop setting

On average, the workshops lasted 1 h and 35 min (range: 1 h and 27 min to 1 h and 48 min). The language of the workshops was Dutch. Each workshop took place in a room with a table large enough to seat five people and to accommodate the A2-sized workshop materials. Data collection took place between June 2024 and December 2025. Workshops 1 and 2 were conducted on 19 and 20 June 2024 with care professionals from care organization A. Following these initial workshops, we decided to expand the organizational sample to examine whether perspectives on social connectedness and technology use extended across different care contexts. Thus, we approached additional care organizations and conducted workshops as these organizations became available to participate. Hence, workshops 3 and 4 took place with care organization B on 27 January 2025 and workshop 5 took place with care organization C on 23 December 2025.

During workshops 1–4, there were two moderators present: one lead moderator and one supporting moderator. The lead moderator guided the exercises and discussions; the supporting moderator took care of distributing and collecting the workshop materials during each phase, took pictures, arranged refreshments, and acted as backup during the discussions. During workshop 5, there was only one moderator present. The first author was the lead moderator in each session; the supporting moderator was an external researcher.

### 3.4 Data collection and analysis

We made audio (Voice Memos app on iOS) and video (GoPro) recordings to enable the production of verbatim transcripts annotated with relevant non-verbal behaviors. The transcripts were generated in Dutch, the native language of the care professionals, with a locally run version of Whisper (55), manually corrected while watching the recordings, and anonymized by replacing participant names with the codes listed in Table 1. The workshop artifacts (i.e., post-it notes, posters, photographs, and schedule maps) were not systematically analyzed as data, but were used to support and structure the workshop discussions and to facilitate participants’ reflection on their experiences.

Data were analyzed following an inductive thematic analysis approach (56), allowing codes and categories to emerge organically from the participants’ accounts. We carried out our analysis following the six-phase framework of thematic analysis originally proposed by (57), while also integrating subsequent refinements and methodological guidance (56). We conceptualized our approach as an iterative process involving continuous movement across phases and as a reflexive practice that foregrounds the researcher’s situated and active role in the construction of meaning (58).

The first and second steps involved data familiarization and initial coding, which were closely intertwined and hence reported together. The first author repeatedly read the transcripts for familiarization while inductively coding the text in English using the qualitative data analysis software MAXQDA (59). As the first author’s first language is Dutch, the coding was conducted directly from the original Dutch transcripts. While coding, the first author periodically grouped codes into provisional, descriptive categories to keep an overview of the analysis and manage the volume of data.

The third, fourth, and fifth stages of the analysis (searching for themes, reviewing themes, and defining and naming themes) were interrelated, iterative, and non-linear (60). After coding the five transcripts, the first author used MAXQDA to review and cross-compare the codes to identify recurring meaningful patterns. Once the initial round of categorization was concluded, the first author re-evaluated each individual code to assess its fit within the existing structure, and then consulted the other authors to review the emerging thematic structure. This process was repeated multiple times, continuously reassessing key takeaways and (sub-)themes in light of their coherence and alignment with the research questions, reflecting a gradual analytic refinement toward themes most relevant to the study. In the final thematic structure, the highest-level groupings served as themes, while lower-level categories comprised sub-themes and narrative elements. For reporting, selected quotations were translated from Dutch into English by the first author. The translations were reviewed by the other Dutch-speaking authors against the original Dutch quotations, and discrepancies were discussed and resolved collaboratively. Particular attention was paid to the preservation of the meaning of language-specific figures of speech, idiomatic expressions, and other linguistic nuances.

Tronto’s Ethics of Care framework (29) did not guide data collection nor serve as an a priori coding scheme during the initial analysis; instead, it was applied retrospectively as an interpretive framework to structure the Discussion and synthesize the broader sociotechnical implications of our findings.

### 3.5 Positionality statement

We recognize that our positionality shaped both the conduct and interpretation of this research (58). All authors have personal experience with dementia through family ties or close relationships, although none have worked as care professionals. Hence, no author has an employment affiliation with the participating care organizations. The last author has experience caring for a family member with dementia. At the same time, all authors have multiple years of experience conducting HCI research with people with dementia across Western care contexts, including the participating care organizations. Across the author team, we adopt a person-centered perspective that foregrounds the retained abilities, agency, and identities of people with dementia rather than deficit-oriented framings (9, 61). This orientation informed both our interpretation of social connectedness and our critical stance toward technologies that may reduce relational care practices to computable representations or standardized interventions.

## 4 Results

Thematic analysis yielded four themes^1^ : (1) care professionals assess social connectedness needs by recognizing behavioral expressions over time; (2) care professionals negotiate social connectedness at the intersection of care practice, regulations, and personal life; (3) technology provides opportunities for social connectedness; and (4) technology use is situated within everyday care.

### 4.1 Care professionals assess social connectedness needs by recognizing behavioral expressions over time

Our data revealed that care professionals perceive social connectedness as important for people with dementia. However, they acknowledge that recognizing social needs in people with dementia is not exactly straightforward. In the earlier stages of dementia, one can rely on the verbal skills of people with dementia to articulate feelings of loneliness or a desire for contact: *“We do have attendees [at daycare] who can express that [social needs] very clearly themselves, like: ‘well, I am really here for my social contact, because I feel lonely when I sit at home alone’ ”* [P8]. However, in later stages of dementia, increasing cognitive and physical impairments make *“expressing those kinds of needs very difficult […] when you are already that far along in your process”* [P3]. Besides, there is often a stigma associated with expressing social needs, involving a *“piece of vulnerability of opening up [about loneliness]”* [P8].

Care professionals agreed that social needs often surface in the form of behavioral responses to social over- or under-stimulation: *“We see a lot of misunderstood behavior, […] and very often it lies in, yes, a social aspect”* [P3]. For instance, social overstimulation as a result of abundant group activities can cause responsive behavior in residents: *“We now have a man, and we strongly suspect autism–so this man is somewhat different in social contact. He was once completely angry. Like: ‘yes, they keep asking me if I want to come play a game, but I do not like that at all. And why do they keep offering that?’ ”* [P11]. Care professionals emphasize that *“some people do not have a need for social contact”* [P11] and stress the importance of allowing people with dementia to recover from social demands, as well as withdraw from them: *“Because I simply saw that people stayed at their level much longer because they had their own room [where they could withdraw]”* [P12].

Understanding where the need for social stimulation of each person with dementia sits is a complex task. Apathy or disengagement can be easily interpreted as a person recovering from overstimulation. However, they might as well be indicators of under-stimulation: *“It is often said, ‘that person is overstimulated.’ But they are also often under-stimulated, and as a result, what remains is, so to speak, that stoic wandering around, for example”* [P3]. While in cases of social under-stimulation, increasing social interaction can be considered helpful, the uncertainty about what is going on with the person with dementia makes care professionals doubtful about what to do, as additional stimuli could both alleviate and exacerbate the situation: *“I find that a challenge, because you can say, that person is under-stimulated, so they need more contact. But maybe they are overstimulated, and that is not something you can just figure out easily”* [P3].

Care professionals reported needing to *“get under someone’s skin”* [P9] to–over time–develop an in-depth understanding of them, enabling them to recognize and interpret responsive behaviors: *“When you get to know someone better, you also learn to pick up on those signals. And when is someone doing well? When they are in balance, when the social, psychological, physical, spiritual–when everything is in balance, that is what you want most. But that is also the biggest challenge, to figure all of that out.”* [P14]. To illustrate how careful observation allows care professionals to infer a social need, P9 shared: *“Yesterday, I had to go to a unit to distribute medication. There is a man there and he had somewhat red eyes. So I went to sit in front of him and said: ‘why do you have red eyes? Do you always have that?’ I said: ‘or have you been crying?’ No answer. I said: ‘do you feel lonely or are you in pain?’ ‘No, I am not in pain.’ Then you find out anyway, right? [Then I draw the conclusion] that at that moment he was lonely and missed his wife.”* Care professionals reported becoming better at distinguishing between over- and under-stimulation this way, by observing people with dementia and continuously assessing how to approach them: *“Do you go in very energetically? Or do you go in very calmly? That is really something you have to sense […] what does this person need from me right now?”* [P3]. They acknowledged that the demeanor of care professionals directly shaped the social atmosphere: *“If you project calmness […], the group will also become calm”* [P7].

### 4.2 Care professionals negotiate social connectedness at the intersection of care practice, regulations, and personal life

Care professionals often perceived themselves as a primary source of social interaction for people with dementia: *“I think that ultimately those who work on the unit are more like family to the clients than their actual family… for the average client”* [P3]. Efforts to foster social connectedness included organizing shared activities such as going *[grocery] shopping together* [P1, P9], *playing games* [P2, P9], *smoking together* [P1, P3, P12], as well as creating environments that invite interaction, such as a *bus stop* [P3] or an *“old men’s bench”* [P3] (i.e., a bench in a central place of the residential care locations where residents can come together). In addition, care professionals identified conversational topics to facilitate everyday conversations, such as *holidays* [P2], *weather* [P7], and *pictures* [P9].

However, addressing social connectedness is positioned in a structurally ambiguous space between formal care obligations, personal moral commitments, and institutional priorities. Within that space, care professionals continuously negotiate whether, how, and to what extent social connectedness can be prioritized.

#### 4.2.1 Negotiation between task-oriented care and relational engagement

Part of the negotiation space lies in whether care professionals adopt a relational or task-oriented approach to social connectedness. From the accounts of care professionals, we should talk about a continuum between relationality and task orientation rather than a dichotomy. The relational approach foregrounded person-centered investment in getting to know the person with dementia and involved an intrinsic motivation to recognize and respond to opportunities for social connectedness. In this context, some described how opportunities for social connectedness could arise in routine care tasks. Moments such as *meal moments* [P12], *administering medication* [P11], and *washing* [P7] were not necessarily experienced as inherently social, but could become so when care professionals deliberately engaged with residents during these activities. For instance, P5 reflected that care moments could be used to initiate meaningful conversations based on personal items in a person’s room: *“You can also do that during care, if there is a photo frame on the nightstand, to start a conversation during care”* [P5]. At the same time, practicing a relational approach to social connectedness is not without personal cost, but requires care professionals to continuously negotiate how much emotional involvement they can sustain. For instance, they could develop strong attachments to residents, making it difficult to disengage after a shift: *“…that client who passed away last week, I find that quite difficult. […] at the moment that she was lying there alone, then I would actually rather not go home. […] imagine if she passes away here alone tonight. […] But yes, I also have a private life [laughs briefly and humorlessly]. So you do take that with you when you go home”* [P2].

On the other hand, a more task-oriented approach could help care professionals maintain clearer personal boundaries and manage the emotional demands of care work, while potentially limiting the extent to which routine care moments are approached as opportunities for social connection. In this context, routine care tasks were not always approached as opportunities for social engagement. Some care professionals focused primarily on the functional completion of care tasks: *“Well, you definitely have care professionals who are purely task-oriented, right. […] Those who are really task-focused… providing functional care: washing… That, and then not saying anything. Closing the door again.”* [P10]. Participants emphasized that people with dementia are highly sensitive to such differences in approach. As P10 reflected, *“… but they [people with dementia] really feel that. And they also say it: […] ‘If that [purely task-oriented] person comes, then I do not need care anymore.’ They sense perfectly who they have in front of them. […] That has everything to do with [social] contact.”*

#### 4.2.2 Negotiation between institutional priorities and personal values

The second part of the negotiation space involved continuously balancing institutional priorities and personal values. The main institutional factors that constrained opportunities to build personal connections with the residents were limited staffing and time constraints. As P11 noted: *“I think that if you can offer those people a fulfilling day, with a fixed structure and also social connectedness, meaningful activities… you can prevent so much. But… care shifts simply do not have time for that. And it is just not feasible.”* Furthermore, different forms of residential care shaped the extent to which person-centered approaches to social connectedness could be implemented. In particular, small-scale living arrangements offered more opportunities for one-to-one interaction, enabling care professionals to better get to know residents and respond to their social needs compared to larger-scale residential care units: *“… it depends on whether you are in a nursing unit or whether you have small-scale living […], like at [location 1; small-scale living]. Of course, they are all people with some form of dementia, but at [location 1], people still walked to [supermarket] themselves. […] [Whereas] when I worked at [location 2; nursing unit], we had a lot of behavioral issues, and that was purely because many people were sitting together in a small living room with eight people, from early morning until late at night. But they were also surrounded by six people whom, in their normal lives, they would probably have avoided by a wide margin”* [P15].

Institutional priorities surrounding safety, health, and risk management further shaped how care professionals navigated opportunities for social connectedness. In some cases, care professionals described deliberately working around or flexibly interpreting organizational policies when these were perceived to constrain socially meaningful interactions for people with dementia. P12 described facilitating an outdoor setting where residents could smoke together: *“A woman does crossword puzzles outside, but she also smokes. Another resident does as well. So they have very nice social contact, with a cigarette outside. Only it is not allowed to be facilitated […]. But then we thought of maybe a heated blanket. And then we frame it as social contact.”* In this way, care professionals sometimes prioritized opportunities for meaningful social interaction over medical advice intended to protect physical health (i.e., not smoking).

While some tensions could be navigated through flexible interpretations of institutional policies, participants also described situations involving social connectedness that raised ambiguous moral and professional dilemmas without clear procedural solutions. For instance, care professionals described situations in which intimacy emerged between residents, requiring careful deliberation about how to respond: ‘P15: *“Or two residents who get into bed together?”* [Everyone laughs] P12: *“Yes, we are actually seriously discussing that proposition right now.”*’ Elaborating on such a situation, P15 described encountering two residents in an intimate setting during an evening shift: *“And then he was sitting in that room with his cane […]. And there was a lady who found him… extremely interesting. […] Then we had lost track of both of them. I thought oh… I walked in and you really saw there, seriously…”* [P15].

Such situations positioned care professionals in dilemmas where they had to balance respect for residents’ autonomy and intimacy with their responsibility to monitor and safeguard well-being. As P12 reflected: *“I was standing at the door […] what do you do then? Because the moment I walk in… I am no longer respecting their intimacy. […] But to what extent are you allowed to infringe on that?”* In addition, decisions about whether and how to document such situations introduced further ambiguity: *“Then, based on your own norms and values, do you report it? And what do you report then? You actually have to report it, because otherwise I cannot do anything with it during the behavioral consultation or the physician’s consultation ”* [P12].

### 4.3 Technology provides opportunities for social connectedness

Care professionals described a broad range of technologies used in residential dementia care, although only some were explicitly associated with fostering social connectedness. We identified three configurations through which care professionals leveraged technology: (1) to facilitate online communication with loved ones, (2) to position technology as the interaction partner, and (3) to facilitate a shared medium for offline interaction between people with dementia and others. Across these configurations, though, care professionals consistently emphasized that technology should be viewed as a complement to, rather than a replacement for human care. *“I do think that human contact will always be necessary to make people’s needs known. And perhaps to be able to put it into practice. Because otherwise it could become very cold and impersonal”* [P7].

#### 4.3.1 Technology for connecting with loved ones

Care professionals repeatedly mentioned their role in using technology to set up an online communication channel between people with dementia and their loved ones at home, thereby allowing for a sense of social connectedness to emerge. They did this, for instance, through video calling: *“The way they, for example, seek contact with other family members, through video calling. We have quite a few women, especially on the psychogeriatric unit, who are really quite active with their iPad”* [P10]. Beyond direct communication, care professionals also envisioned technologies that could help people with dementia maintain a sense of ongoing presence and social connectedness with loved ones, for instance through continuously sharing photos or updates with relatives: *“I would really like many people to get a digital photo frame. You can just send a photo every time”* [P9].

#### 4.3.2 Technology as an interaction partner

In a few other cases, care professionals mentioned having tried out technologies that took an active role as an interaction partner of people with dementia, for instance a social robot that initiated engagement: *“We already have a robot that indeed says… ‘Hello, shall we play a game?’ ‘Okay.’ And it initiates that”* [P7]. While social robots had occasionally been introduced into care practice, participants also reflected on emerging forms of conversational AI as potential future interaction partners for people with dementia. P7 described their own experience with ChatGPT as resembling interaction with another person, and speculated how such technology could provide a sense of meaningful contact in situations of loneliness associated with dementia: *“I discovered ChatGPT, where I really feel like I am communicating with a person. […] It feels like personal contact. […] Suppose I am lonely […] and I simply get contact… then I would not see a problem with that”* [P7].

#### 4.3.3 Technology as a shared medium for social interaction

Finally, care professionals described technologies that functioned as a shared medium for in-person social interaction between people with dementia and others (e.g., care professionals, fellow residents, or loved ones), ranging from everyday activities such as listening to music or watching television together to more interactive systems. For instance, care professionals used an interactive table that could be used for *“playing games, […] [playing] from the 50s, 60s, 70s, 80s, […] [practicing] sayings… proverbs…”* [P15] together with people with dementia: *“We also used it a lot […] for people who, for example, could not leave their apartment for a few days. So that you would just do something one-on-one”* [P15]. Participants particularly valued projection-based interactive systems for their ability to stimulate collective engagement among residents, which led some care organizations to install additional systems in shared living spaces: *“We recently had an extra [projection-based interactive system] installed in the living room. So that it [gets used] more…”* [P14].

### 4.4 Technology use is situated within everyday care

Care professionals described technology use for social connectedness as limited and largely reactive within everyday care practice.

#### 4.4.1 Technology remains peripheral in everyday care

Overall, participants reported that technologies with a social purpose were often not used consistently, and they provided several reasons for this. First, there was a lack of awareness of the kinds of technologies that are readily available in the care organization: ‘P12: *“Yes, but that is it, right. That people often do not even stop to realize that it is there.”* P13: *“And recently I brought it up, ‘oh, I did not even know we had that.’ ”* P14: *“Missed opportunity.”*’ Even when there was awareness of certain types of technologies, their use was not guaranteed. Care professionals reported that a lack of knowledge of or confidence to operate certain systems caused technologies for social connectedness to remain unused in practice: *“I went to a well-being staff member and I said, ‘but why are you not using that thing?’ I said, ‘in your role…’ [Whispers] ‘We do not know how it works’.”* [P15]. A similar lack of knowledge or confidence was projected onto people with dementia, suggesting that technologies might become more relevant for future generations of people with dementia who have had greater exposure to technology throughout their lives: *“Yes, I immediately pictured such a robot driving around. You also have restaurants where they already… It might be more for the later [upcoming] generations”* [P5].

Another reason why technologies for social connectedness remained weakly embedded in everyday care practice was that care professionals were not always meaningfully involved in organizational decisions surrounding their introduction: *“I arrived at the unit there, so I first did an interview, and then that care assistant said, ‘that interactive table, we do not use it, […] [because] we did not ask for it! Plus, our team manager thinks, then at least they have such an interactive table. Well, because I was not involved in it, I do not use it!’ ”* [P15]. Furthermore, adopting technology often requires deviating from established routines, which can be difficult in practice: *“And of course you notice, the longer you have worked in a certain field… ‘this is how we have always done it,’ it also becomes harder to deviate from that”* [P7]. At the same time, participants noted that resistance to new technologies is not fixed, but can shift through exposure and experience, as illustrated by earlier transitions toward digital systems in care: *“I remember, in the past we had to switch from paper to computer. [Adopts a dramatic tone] ‘Oh, that computer…’ […] so much resistance. And after two weeks, everyone was using it.”* [P2].

Participants further described practical and logistical constraints that limited the integration of technologies for social connectedness into everyday care routines. For example, some technologies had fixed locations that required care professionals to move residents or relocate equipment [P2, P13], while others were only available on specific units and therefore required coordination with colleagues before they could be used [P5]. As a result, whether and how such technologies are used largely depends on individual care professionals’ initiative, competence, and ability to integrate them into ongoing care routines.

#### 4.4.2 Technologies are primarily used reactively to observed behavior

The peripheral positioning of technologies for social connectedness in everyday care practice shaped how and when they were used. Rather than being proactively integrated into ongoing relational care, technologies were typically leveraged in response to observable signs of responsive behavior in people with dementia. That is, participants reported a desire to deploy technologies when a person with dementia became restless: *“you just want to be able to seize the moment when someone is restless and then activate something”* [P5].

However, their use often depended on individual care professionals recognizing opportunities for meaningful use in the moment. This became particularly visible in the cases where technologies did become integrated into care practice, often driven by intrinsically motivated ‘technology ambassadors’ who actively promoted and facilitated their use among colleagues. As P11 commented: *“And you need a driver, right? So we now have [inaudible] with the [name] robot. But in the beginning, nothing was done with it. It was sitting somewhere in the corner and occasionally, ‘oh yes, there it is.’ But at some point there was an active aging nurse, and she was very engaged with it. ‘And oh, would it be something for that person, something for that person.’ And now it is running. Because now people know, ‘oh, wait, we have that robot, we can set something up with it.’ And then she created a ready-made setting. So the only thing you have to do is place it and press that button.”* [P11]. Intrinsic motivation and enthusiasm for technologies were described as the key to successful adoption. If such an ambassador was appointed without that intrinsic motivation, the successful integration of technologies into care practice remained uncertain: *“We say that we have care technology ambassadors in the organization… but what knowledge are we actually giving those people? None. Are people even intrinsically motivated or… I think a third were simply put forward by the team, like, ‘well, you just do it’ ”* [P15].

## 5 Discussion

This study investigated how care professionals in residential care understand and foster social connectedness in people with dementia (RQ1), as well as how digital technologies could fit within these practices (RQ2). Our findings position social connectedness in dementia care not as a stable state to be detected or delivered, but as a situated relational achievement that emerges through ongoing interpretive care practices.

To interpret our findings and answer RQ2, we adopt Tronto’s (29) ethics of care framework. Here, we use Tronto’s (29) ethics of care framework as an interpretive lens to consider how social connectedness is interpreted, negotiated, enacted, and evaluated by care professionals. For each of the phases of Tronto’s (29) ethics of care framework, we discuss the relevant empirical findings related to social connectedness and the use of technology, and develop ethical and sociotechnical implications for responsibly introducing digital technologies where supported by our findings.

### 5.1 Caring about

In terms of noticing a need for social connectedness, our findings show that identifying the social needs of people with dementia increasingly relies on non-verbal communication the more the disease progresses. In line with prior work (35, 36, 62, 63), our findings confirm that social needs are typically expressed by people with dementia through responsive behaviors and ascribed by care professionals to social over- and under-stimulation. However, our findings further show that translating responsive behavior into social needs is not a straightforward process, but requires a person-centered approach, as unmet social needs do not reside in a specific behavior. Rather, the meaning of a particular behavior might differ across individuals with dementia. In residential contexts, this entails that care professionals require profound knowledge of a person’s history and behavioral repertoire to infer a social need, and aligns with person-centered care’s emphasis on seeing people with dementia as individuals with unique histories, preferences, and relational needs rather than through the lens of their cognitive decline (9).

Our findings do not speak directly to the use of digital technologies for recognizing social needs. However, they highlight important considerations for the hypothetical positioning of such technologies in this phase of care. Our empirical results show that care professionals recognize a need for social connectedness through their ongoing relationships with residents, allowing them to interpret subtle behavioral changes in light of their knowledge of the individual and the situation. This makes social needs recognition a highly situated and continually updated process (49, 64). In contrast, digital technologies designed to support the recognition of social needs typically rely on observable signals that are separated from the relational and situated context in which they acquire meaning. As (65, p. 148) argues, computational systems *“necessarily reduce complexity, and (…) remove significant context, in order to make the world more computable”*. Applying such technologies to the recognition of social needs could therefore risk reducing behavior that is meaningful within a particular care relationship to a decontextualized signal. Moreover, introducing technology into this process may create new relational tensions and vulnerabilities within care relationships (52). If digital technologies were to assume or substantially support the recognition of social needs, they could potentially help care professionals identify needs that might otherwise remain unnoticed. At the same time, such support could create a dependency on technological assessments, reducing opportunities for care professionals to invest in the ongoing relationships through which they develop the knowledge and sensitivity needed to interpret residents’ behavior, and ultimately perform their job. This could, in turn, undermine the relational value embedded in the process of recognizing social needs itself and lead to deskilling. This reflects what (66, p. 117) describes as the *“doorman fallacy”*: replacing a role based on its apparent function while overlooking the implicit social and relational value embedded in its performance. Technology may thus simultaneously support the recognition of social needs while undermining the relational processes through which those needs become recognizable in the first place, illustrating its double-edged nature (50, 51).

**Implication 1**: Digital technologies should not be positioned as means to independently recognize social needs, as they risk undermining the relationship-building through which care professionals develop the knowledge needed to foster social connectedness. Instead, digital technologies could support care professionals in identifying potential indicators of social needs, without replacing the relational judgment required to interpret them.

### 5.2 Taking care of

In terms of identifying the right responses to address needs for social connectedness, care professionals described continuously negotiating possible responses based on the preferences of people with dementia, their caregiving styles, available time and staffing, and broader institutional routines and norms. Deciding how to foster social connectedness appeared to be a situated, person-centered process, shaped by both relational considerations and the practical realities of residential care (30, 48). Rather than following a fixed set of responses, care professionals therefore adapt what they do to the person, the situation, and the possibilities available to them. This positions *taking care of* not simply as deciding what action to take, but as continuously negotiating which response may be meaningful and feasible in a particular situation.

Our findings suggest that digital technologies currently form only a peripheral part of this repertoire of possible responses. Technologies were rarely described as part of the process of deciding how to foster social connectedness, in part because care professionals were not always aware of the technological opportunities available to them, but also because they did not always feel confident using particular systems, and were not necessarily involved in decisions about which technologies were introduced into their organization. These findings align with prior work showing limited awareness of available technologies in dementia care (67), a tendency for interventions to be adopted reactively rather than being embedded within everyday care routines (68), and a lack of integration of technologies into existing workflows and routines (69). Specifically, our findings show that technology use was described as occasional and tailored to specific situations, depending on whether professionals knew about a particular digital technology, could use it, and could incorporate it into the situation at hand.

**Implication 2**: Digital technologies should be positioned as resources that expand, rather than prescribe, care professionals’ repertoire of responses available for tailoring and exploring methods for fostering social connectedness. Their use should preserve care professionals’ situated judgment and responsibility for deciding which response is appropriate. Introducing digital technologies into the process of deciding how to address a need for social connectedness comes with a caveat: technology use itself may imply new norms, elicit and structure new behavior, and gradually make such technology indispensable. This could lead to *“moral tunneling”.* It may reshape and narrow which social connectedness responses are considered, foregrounding generalized suggestions over more nuanced and context-sensitive possibilities that emerge in practice (70).

### 5.3 Care giving

In terms of targeting social needs in care practice, our findings show that care professionals work within busy care routines and tightly structured daily schedules, where attention tends to be directed toward fulfilling residents’ primary care needs rather than specifically addressing their social needs. This is in line with prior work showing that staff shortages and time constraints frequently lead care priorities to center on functional care at the expense of social and psychosocial needs (14, 71, 72), despite growing recognition of the importance of prioritizing psychosocial needs in dementia care (73).

Nevertheless, we found that care professionals occasionally used technology to support the enactment of social connectedness in one of three ways: (1) facilitating online communication with loved ones, (2) positioning technology as the interaction partner, and (3) establishing a shared medium for offline interaction with others. These configurations broadly resonate with the functional triad of technology roles (74), which positions them as tools (i.e., carrying out a task), media (i.e., structuring interactive experiences), or social actors (i.e., stimulating social interaction). Note that these roles are not mutually exclusive, but their boundaries may blur in practice. When technologies are used by care professionals, the roles they take are not merely dependent on their functional design, but rather on how they are operationalized in practice. For instance, a reminiscence tool [e.g., (41)] can be used for individual use, positioning it as a tool (for memory retrieval) and a social actor (for discussing the memory), but also for group use, positioning it as a medium for shared interaction. Our findings suggest that the social value of these roles does not reside in their epistemological differences, but rather in how they *mediate* meaningful interaction between people with dementia and others, which is determined by how care professionals integrate them into ongoing relational practices. Across these configurations, participants described technology as facilitating particular forms of contact, interaction, or shared activity, rather than as replacing the human relationships involved in fostering social connectedness. This was also reflected explicitly in participants’ emphasis on the continued importance of human contact.

**Implication 3**: Digital technologies should therefore be positioned in ways that allow tool-, medium-, and social actor-like configurations to mediate and support meaningful interaction between people with dementia and others rather than substitute such interaction. Practically, this suggests opportunities for digital technologies that facilitate communication with loved ones, scaffold shared activities, support co-creation and shared meaning-making, or help care professionals initiate and sustain meaningful interaction around everyday care practices. However, as digital technologies increasingly acquire human-like conversational capacities, for instance through advances in Generative AI (75), there is a risk that they become positioned as autonomous interaction partners, thereby redistributing relational work away from caregivers, loved ones, and peers toward interaction with computational systems themselves. In this context, social connectedness risks becoming reduced to an interactional exchange that can be computationally simulated rather than relationally lived (29, 64).

### 5.4 Care receiving

The final phase of care turns attention to how care is received and whether the needs of the person receiving care have been met (29). Our findings show that, in line with person-centered care (9), care professionals interpret subtle behavioral, emotional, and relational changes to assess whether social needs have been met. Typically, moments of calmness, reciprocal engagement, participation in interaction, or the disappearance of responsive behavior are indications that social interaction is aligned with the person’s needs in that moment. At the same time, our findings show that interpreting such indications is an ambiguous process. For instance, withdrawal or calmness may indicate that a person does not require further social stimulation, but may also reflect other circumstances, such as recovery from social overstimulation. This makes it difficult to treat observable behavior as an indication that a social need has or has not been met without situated contextualization.

This ambiguity raises a broader consideration for the potential use of digital technologies to assess residents’ responses to social interaction. Technologies that rely on observable behavioral or affective indicators risk treating context-dependent and individually meaningful responses as standardized signals whose meaning can be computationally determined (26). Speculating on the prospective use of such technologies calls for caution when inferring social states from behavioral measures alone so as not to omit contextual factors in the interpretation of a person’s response to social interaction.

### 5.5 Limitations and future work

Our study has several limitations. First, our study relied exclusively on care professionals’ perspectives. As a result, our findings describe how care professionals interpret and respond to social connectedness, but cannot establish whether these interpretations correspond to the subjective experiences or preferences of people with dementia. Second, because participation was voluntary, our sample likely overrepresents care professionals with greater interest in relational care and technology use. Third, the findings are situated within the organizational and cultural context of residential dementia care in The Netherlands, limiting direct transferability to other care systems and contexts. Fourth, our study focused exclusively on residential care, while most people with dementia, at least in The Netherlands, live at home, where social connectedness is enacted differently and care professionals play a less central role. Future research should therefore include more diverse care professionals and care contexts, particularly home care settings involving people with dementia themselves and, where appropriate, their relatives or advocates.

## 6 Conclusion

This study is an empirical exploration of the perspectives of care professionals working with people with dementia on social connectedness (RQ1) and the sociotechnical role of conventional digital technologies in residential dementia care (RQ2). It uses ethics of care as a moral lens to evaluate the use of digital technologies during different phases of fostering social connectedness in people with dementia. Findings from workshops with care professionals suggest that fostering social connectedness is a person-centered, relational process at the intersection of personal and institutional values and constraints. In the context of fostering social connectedness, digital technologies played a limited but varied role, supporting communication, interaction, and shared activities, while remaining largely peripheral to everyday care practices. Drawing on Tronto’s ethics of care framework, we considered the four phases of care as four interconnected phases of fostering social connectedness in people with dementia. Across these phases, our findings suggest that the value of care lies not only in whether social needs are ultimately addressed, but also in the relational processes through which care professionals come to recognize, negotiate, enact, and interpret how care is received in practice. From this perspective, the use of digital technologies at different phases of fostering social connectedness raises ethical and sociotechnical questions that extend beyond concerns about technical performance alone. While digital technologies may support care professionals in fostering social connectedness, our findings suggest that such systems should not replace the relational processes through which social connectedness emerges in practice. Rather, digital technologies may be more ethically appropriate when positioned as a mediating resource that supports shared interaction and relationship-building between people with dementia, care professionals, and loved ones. In this sense, the ethical implications of digital technologies in dementia care concern not only what these systems can do, but also how they may reshape the relational practices through which meaningful social connectedness is understood and enacted.

## Data Availability

The raw data supporting the conclusions of this article will be made available by the authors, without undue reservation.

## Footnotes

^1^Digitalized versions of the filled-out workshop materials can be found on OSF: https://lnk.ua/lnmT9p2MV
